# Prognosis of Out‐of‐Hospital Cardiac Arrest in Underserved Rural Area

**DOI:** 10.1002/clc.70059

**Published:** 2025-02-03

**Authors:** Dor Ezon, Hagay Shwartz, Sagi Gleitman, Zeev Israeli, Asaf Miller, Edo Y. Birati

**Affiliations:** ^1^ Azrieli Faculty of Medicine Bar‐Ilan University Safed Israel; ^2^ Tzafon Medical Center, The Kittner‐Davidai Division of Cardiovascular Medicine Poriya Israel; ^3^ Medical Intensive Care Unit, Rambam Medical Center Haifa Israel

**Keywords:** advanced cardiac life support, outcome, out‐of‐hospital cardiac arrest, prognosis, rural area

## Abstract

**Background:**

Epidemiological data are lacking on patients in the rural areas who are being admitted after out‐of‐hospital cardiac arrest (OHCA). We report here the first descriptive analysis study of patients who were hospitalized and treated after OHCA at an academic medical center in the Lower Galilee which located in the north part of Israel.

**Methods:**

This is a descriptive, retrospective analysis of all patients admitted after OHCA to Tzafon Medical Center between the years 2017 and 2023. The analysis consists of the epidemiological, social, and clinical data based on the electronic medical records.

**Results:**

A total of 62 patients were included in this analysis, 82% were men with a median age of 61.5 years old. Thirty‐four percent had history of ischemic heart disease (IHD) and 60% history of hypertension. Twenty‐seven (44%) patients died during their admission. In 49 (79%) patients the first rhythm on emergency medical service (EMS) arrival was a shockable rhythm and 30 (48%) patients had a ST‐elevation myocardial infarction (STEMI) on electrocardiogram (ECG). Patients who were admitted to the hospital after OHCA were more likely to be discharged alive if they had no history of IHD (*n* = 27; *p* = 0.037), hypertension, or hyperlipidemia. Moreover, the presence of first shockable rhythm on the first ECG that performed by EMS was associated with higher rates of survival (*n* = 33; *p* < 0.001).

**Conclusions:**

We present the first single‐center epidemiological analysis of patients admitted after OHCA at a rural area in Israel, with an in‐hospital survival rate of 56%. Patients without history of IHD, hypertension, hyperlipidemia, and acute kidney injury and those with first shockable rhythm were more likely to discharged alive.

## 1 Introduction

Out‐of‐hospital cardiac arrest (OHCA) is a medical emergency with high morbidity and mortality rates [1]. The prevalence of OHCA varies globally. According to current data, Asia exhibited the lowest incidence rate (52.5/100 000 person‐years) compared to nearly double the incidence rate in North America (98.1/100 000 person‐years), Europe (81.6/100 000 person‐years), and Australia (112.5/100 000 person‐years) [2]. Return of spontaneous circulation (ROSC) and overall survival in OHCA were found to be independently related to the time from event to initiation of effective chest compressions, time to defibrillation in patients with shockable rhythm, and other factors such as the cause of the event and comorbidities [3].

Israel has made a significant progress in enhancing the provision of prehospital care and promoting public access to early defibrillation through the deployment of automated external defibrillators (AEDs) [4]. However, despite these efforts, OHCA remains a significant public health concern with substantial regional disparities [5]. The outcome of OHCA in remote areas was found to be lower than in urban areas although it is found that rural communities exhibited higher rates of bystander cardiopulmonary resuscitation (CPR) [6, 7]. In addition, the rates of OHCA were higher in young rural patients compared to young metropolitan residents, however, the causes of cardiac arrest were similar [6].

The prehospital practice protocol in the region served by the Tzafon Medical Center (TMC) involves several key components to ensure rapid and effective response to OHCA cases. EMS personnel, provided by the single national EMS service Magen David Adom (MDA), are trained to recognize and promptly respond to OHCA incidents. For each OHCA case, the nearest available ambulance, first responders, and a mobile intensive care unit (MICU) are immediately dispatched. Protocols include the immediate initiation of CPR, use of AEDs, and advanced life support (ALS) measures when necessary. CPR is performed in every OHCA case, except in cases where death is certain (e.g., signs of decay, organ failure) or where the patient has a do‐not‐resuscitate (DNR) order. EMS teams coordinate closely with the TMC to provide continuous care during patient transport, ensuring a seamless transition from the prehospital setting to the emergency department.

Israel has two distinct rural areas: the north and southern regions. The Lower Galilee area is an 800 km^2^ region with distinct geographic characteristics within northern Israel with a population of around 13 400 people, known for its expansive agricultural landscapes and scattered communities. The patient profile typically includes a diverse demographic, with a mix of agricultural workers, small‐business owners, and retirees. The region is characterized by relatively sparse population density for the metropolis and limited access to specialized healthcare services. The Lower Galilee is served by one hospital, the TMC, which located in the one of the largest cities in the Lower Galilee, Tiberias, and is about 100 km away from the nearest metropolis (Haifa). As there is no study examining OHCA in rural Israel, the features of the Lower Galilee present a valuable opportunity to understand the gaps and challenges of this entity.

This study focuses on the epidemiological characteristics of OHCA patients in a rural area in Israel. By conducting a retrospective analysis of patient records, we aim to shed light on the unique aspects and disparities of OHCA in this remote region.

## 2 Materials and Methods

### 2.1 Study Population

This study included all patients admitted to the TMC intensive coronary care unit (ICCU) after an OHCA with a suspected cardiac origin between January 2017 to December 2023. During the study period, the TMC accepted all emergency patients presenting with OHCA within the possible radius of the hospital. Given the urgency of these cases, TMC was the closest and safest facility for patients to receive immediate and appropriate care. Data were collected from the hospital electronic medical records. The database consists of the epidemiological and clinical data of all patients with OHCA. Patients who died in the emergency room were included in the overall analysis of OHCA outcomes. Their data were analyzed alongside other patient outcomes to provide a comprehensive understanding of the prognosis in these cases. Key metrics such as interventions performed and clinical history were recorded and evaluated. Exclusion criteria were age under 18 years and OHCA from noncardiac origin. We extracted the following data from the database: age, gender, race, history of heart failure, history of ischemic heart disease (IHD), presence of atrial fibrillation, hypertension, hyperlipidemia, or diabetes mellitus, the first rhythm (shockable or not), history of acute kidney injury, amines administrations during resuscitation, medical and interventional therapy during admission (including inotropic and pressor therapy, mechanical support, percutaneous transluminal coronary angioplasty [PTCA], etc.). Patients were divided into two groups: discharged alive or dead during admission.

### 2.2 Statistical Analysis

Categorical variables were reported with frequencies and percentages and continuous variables as medians with interquartile range. We used the *t*‐tests for continuous parameters and the *χ* ^2^ test for categorical variables. A *p *< 0.05 was considered statistically significant. We used all the available data from our databases within the study time frame. Missing data were handled using listwise deletion. Data analysis was conducted with IBM SPSS statistics version 27.0.1.0.

### 2.3 Ethical Approval

The study was approved by the Institutional Review Board (TMC). The need for written informed consent was waived due to the retrospective nature of the study.

## 3 Results

**Figure 1:**
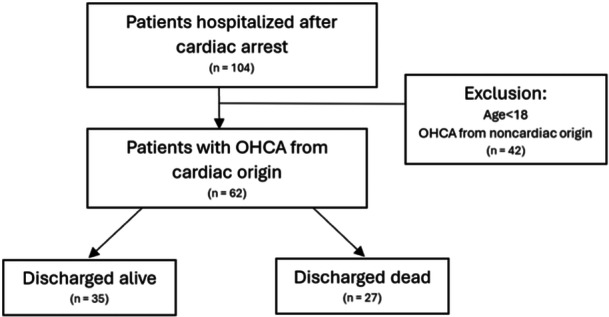
Study patients flow. OHCA, out‐of‐hospital cardiac arrest.

A total of 104 patients admitted and hospitalized with a diagnosis of cardiac arrest were initially identified for this study. After applying the study criteria, 62 patients were included in the final analysis (Figure 1). The baseline characteristics are summarized in Table 1. The median age was 61.5 years old, 82% were men, and 34% had a history of IHD.

**Table 1:** Patients baseline characteristics.

Characteristic	Total (*n* = 62)	Discharged alive (*n* = 35)	Discharged dead (*n* = 27)	*p* value
Age, year	61.5 (53.3–71.8)	59.0 (52.5–64.5)	67.0 (56.0–80.0)	0.144
Gender (male)	51 (82%)	30 (86%)	21 (78%)	0.417
History of heart failure	10 (16%)	6 (17%)	4 (15%)	0.805
History of ischemic heart disease	21 (34%)	8 (23%)	13 (48%)	0.037
Atrial fibrillation	4 (6%)	3 (9%)	1 (4%)	0.439
Hypertension	37 (60%)	17 (49%)	20 (74%)	0.042
Hyperlipidemia	38 (61%)	17 (49%)	21 (78%)	0.019
Diabetes mellitus	15 (24%)	6 (17%)	9 (33%)	0.140

*Note:* Values are presented as medians with interquartile range or number (percentage). All differences were considered significant at a probability level of 95% (*p* < 0.05).

Twenty‐seven (44%) patients died during their admission. The median length of hospitalization of patients was 8 days. In 49 (79%) patients the first rhythm on EMS arrival was a shockable rhythm and 30 (48%) patients had a STEMI on ECG. The variables during the admission of the patients are described in Table 2. Patients with a history of hypertension and hyperlipidemia were more likely to die during the admission (49% vs. 74%, and 49% vs. 78%, respectively, *p* = 0.042 and *p* = 0.019), and that was statistically significant. Moreover, patients who died during their admission were more likely to have a history of IHD (23% vs. 48%; *p* = 0.037) and had higher rates of acute kidney injury (29% vs. 59%; *p* = 0.015). In addition, patients with a first shockable rhythm were more likely to be discharged alive (94% vs. 59%; *p* < 0.001). The median time from the arrival of EMS to PTCA was shorter among patients who were discharged alive (51 vs. 120 min, *p* < 0.001).

**Table 2:** Variables during admission.

Characteristic	Total (*n* = 62)	Discharged alive (*n* = 35)	Discharged dead (*n* = 27)	*p* value
Total hospitalization days	8.0 (5.0–12.5)	10.0 (6.0–14.0)	5.0 (1.0–10.0)	0.032
Time to PTCA (minutes)	—	51 (25‐65)	120 (60–146)	< 0.001
STEMI on ECG	30 (48%)	21 (60%)	9 (33%)	0.051
First shockable rhythm	49 (79%)	33 (94%)	16 (59%)	< 0.001
Acute kidney injury	26 (42%)	10 (29%)	16 (59%)	0.015
Amines administration	41 (66%)	21 (60%)	20 (74%)	0.246

*Note:* Values are presented as medians with interquartile range or number (percentage). All differences were considered significant at a probability level of 95% (*p* < 0.05).

Abbreviations: ECG, electrocardiogram; PTCA, percutaneous transluminal coronary angioplasty; STEMI, ST‐elevation myocardial infarction.

## 4 Discussion

This study describes the unique patient population presenting with out‐of‐hospital cardiac arrest in a rural underserved area in Israel. According to our results, patients who were admitted after OHCA from a cardiac origin are more likely to be discharged alive if they had no history of IHD, hypertension, hyperlipidemia, or had an acute kidney disease. Also, the presence of first shockable rhythm in the first ECG that performed by EMS was associated with higher rates of survival [8].

Several comorbidities and prognostic factors influencing OHCA have already been studied. In their recently published study, Han et al. showed that the first shockable rhythm increased survival rates and was used as a positive prognostic factor [9]. These findings, which emerged in these studies, correspond with our results.

Moreover, hypertension, a prevalent cardiovascular condition, significantly impacts OHCA outcomes according to our findings. Previous studies indicate that individuals with hypertension are more prone to experiencing OHCA, and this condition can exacerbate the severity and complexity of resuscitation efforts. A study by Sonoda and colleagues on myocardial infarction patients highlighted that traditional coronary risk factors play a crucial role in the incidence and prognosis of OHCA [10].

Our results also demonstrate that history of hyperlipidemia is also associated with poorer outcomes in OHCA patients. A systematic review and meta‐analysis published in the *Cureus Journal of Medical Science* by Reyaz and colleagues analyzed various prehospital prognostic factors, including hyperlipidemia, and found that it is significantly associated with the incidence of OHCA [11] and it can be seen that it is consistent with the results of our study.

Conducting the current study is important in understanding the unique needs and risk factors of this patient population. For example, our results demonstrate that patients who survived had a shorter time from admission to coronary intervention. Recent study showed no significant benefit for early versus delayed catheterization in OHCA patients with no ST elevation on postresuscitation ECG [12]. However, it remains unclear whether certain subgroup of OHCA patients would still benefit from early intervention.

Our group showed on a previous study that early recognition of cardiac symptoms is essential to improve outcomes. A cohort of 1810 OHCA patients with witnessed collapse had better outcomes and improving the public awareness of cardiac symptoms and early call for EMS services was shown to be beneficial in an effort to increase survival of this patient population [13, 14]. This may be even more crucial in the underserved areas, as reflected in the current study [15]. Also, because of the lower socioeconomic status and health disparities in the periphery, it is likely that this patient population will have poorly controlled cardiac risk factors and higher rates of tobacco use. Furthermore, it is likely that these socioeconomic differences between the periphery and the metropolis also affect the outcomes of OHCA in one way or another, therefore we believe that further research is necessary to examine these differences in depth and perhaps formulate strategies to improve the survival of the population lives in rural areas. Beyond that, it should be taken into account that the distances from the medical centers in the periphery areas are longer. Although our current study primarily focuses on the prognosis of OHCA in an underserved rural area, we acknowledge that neurological outcomes are a critical aspect of post‐OHCA recovery. To address this, we are planning a supplementary study based on the same database, specifically examining neurological outcomes. This planned study will track patients over a longer period to assess their neurological status using standardized tools such as cerebral performance category (CPC) scale and clinical test results. We aim to identify factors that influence neurological recovery and long‐term quality of life. We believe this additional research will provide a more comprehensive understanding of patient outcomes and inform future interventions.

Our study has several limitations. Although our data represent the “real world” of rural underserved area, our data consist of a single‐center population. Moreover, our cohort is relatively small and may be under‐powered to identify more prognostic factors. It is also important to note that the findings may not fully generalize to other regions due to variations in legislation and emergency medical systems worldwide. This contextual limitation should be considered when applying our findings to broader clinical practice. Furthermore, a detailed analysis of neurological outcomes is planned for future studies based on our database.

In conclusion, we present the current data on risk factors and prognosis of OHCA patients in the rural area in Israel. Future studies are needed to assess strategies on improving the survival of this unique patient population.

## Ethics Statement

This study was performed in accordance with the Declaration of Helsinki and approved by the Tzafon Medical Center Review Board (IRB# 0076‐23‐POR, approved on 2023‐11‐26).

## Consent

The study used anonymized archived data, with no direct involvement of individuals in any procedures or investigations. Therefore, informed consent was waived.

## Conflicts of Interest

The authors declare no conflicts of interest.

## Data Availability

Data are contained within the article.

## References

[1] M. S. Link , L. C. Berkow , P. J. Kudenchuk , et al., “Part 7: Adult Advanced Cardiovascular Life Support,” Circulation 132, no. 18 (2015): S444–S464, 10.1161/CIR.0000000000000261. 26472995

[2] J. Berdowski , R. A. Berg , J. G. P. Tijssen , and R. W. Koster , “Global Incidences of Out‐of‐Hospital Cardiac Arrest and Survival Rates: Systematic Review of 67 Prospective Studies,” Resuscitation 81, no. 11 (2010): 1479–1487, 10.1016/j.resuscitation.2010.08.006. 20828914

[3] L. J. Morrison , R. W. Neumar , J. L. Zimmerman , et al., “Strategies for Improving Survival After In‐Hospital Cardiac Arrest in the United States: 2013 Consensus Recommendations,” Circulation 127, no. 14 (2013): 1538–1563, 10.1161/CIR.0B013E31828B2770. 23479672

[4] M. Ringh , M. Rosenqvist , J. Hollenberg , et al., “Mobile‐Phone Dispatch of Laypersons for CPR in Out‐of‐Hospital Cardiac Arrest,” New England Journal of Medicine 372, no. 24 (2015): 2316–2325, 10.1056/NEJMOA1406038/SUPPL_FILE/NEJMOA1406038_DISCLOSURES.PDF. 26061836

[5] C. Sasson , M. A. M. Rogers , J. Dahl , and A. L. Kellermann , “Predictors of Survival From Out‐of‐Hospital Cardiac Arrest,” Circulation: Cardiovascular Quality and Outcomes 3, no. 1 (2010): 63–81, 10.1161/CIRCOUTCOMES.109.889576. 20123673

[6] G. A. Peters , A. J. Ordoobadi , A. R. Panchal , and R. E. Cash , “Differences in Out‐of‐Hospital Cardiac Arrest Management and Outcomes Across Urban, Suburban, and Rural Settings,” Prehospital Emergency Care 27, no. 2 (2023): 162–169, 10.1080/10903127.2021.2018076. 34913821

[7] P. Nikonowicz , R. Huebinger , R. Al‐Araji , et al., “Rural Cardiac Arrest Care and Outcomes in Texas,” American Journal of Emergency Medicine 78 (2024): 57–61, 10.1016/J.AJEM.2023.12.033. 38217898

[8] B. Grunau , J. C. Reynolds , F. X. Scheuermeyer , et al., “Comparing the Prognosis of Those With Initial Shockable and Non‐Shockable Rhythms With Increasing Durations of CPR: Informing Minimum Durations of Resuscitation,” Resuscitation 101 (2016): 50–56, 10.1016/J.RESUSCITATION.2016.01.021. 26851705

[9] K. S. Han , S. W. Lee , E. J. Lee , M. H. Kwak , and S. J. Kim , “Association Between Shockable Rhythm Conversion and Outcomes in Patients With Out‐of‐Hospital Cardiac Arrest and Initial Non‐Shockable Rhythm, According to the Cause of Cardiac Arrest,” Resuscitation 142 (2019): 144–152, 10.1016/J.RESUSCITATION.2019.07.025. 31377392

[10] T. Sonoda , H. Wada , M. Ogita , et al., “Clinical Features and Predictors of Outcome in Patients With Acute Myocardial Infarction Complicated by Out‐of‐Hospital Cardiac Arrest,” BMC Cardiovascular Disorders 22, no. 1 (2022): 185, 10.1186/S12872-022-02628-3/FIGURES/3. 35439919 PMC9020007

[11] I. Reyaz , C. R. Wei , A. Rawat , et al., “Predictors of Out‐of‐Hospital Cardiac Arrest in Patients Hospitalized With Acute Coronary Syndrome: A Systematic Review and Meta‐Analysis,” Cureus 15, no. 11 (2023): e48609, 10.7759/CUREUS.48609. 38084190 PMC10710753

[12] S. Desch , A. Freund , I. Akin , et al., “Angiography After Out‐of‐Hospital Cardiac Arrest Without ST‐Segment Elevation,” New England Journal of Medicine 385, no. 27 (2021): 2544–2553, 10.1056/NEJMOA2101909/SUPPL_FILE/NEJMOA2101909_DATA-SHARING.PDF. 34459570

[13] E. Y. Birati and A. Roth , “Telecardiology,” Israel Medical Association Journal 13, no. 8 (2011): 498–503, 10.5005/jp/books/12785_174. 21910377

[14] E. Y. Birati , N. Malov , Y. Kogan , et al., “Vigilance, Awareness and a Phone Line: 20 Years of Expediting CPR for Enhancing Survival After Out‐of‐Hospital Cardiac Arrest,” Resuscitation 79, no. 3 (2008): 438–443, 10.1016/J.RESUSCITATION.2008.08.002. 18952353

[15] N. Goldberger and Z. Haklai , “Mortality Rates in Israel From Causes Amenable to Health Care, Regional and International Comparison,” Israel Journal of Health Policy Research 1, no. 1 (2012): 41, 10.1186/2045-4015-1-41/FIGURES/7. 23098080 PMC3502080

